# Predicting Circulatory Diseases from Psychosocial Safety Climate: A Prospective Cohort Study from Australia

**DOI:** 10.3390/ijerph15030415

**Published:** 2018-02-27

**Authors:** Harry Becher, Maureen F. Dollard, Peter Smith, Jian Li

**Affiliations:** 1 Asia Pacific Centre for Work Health and Safety, A WHO Collaborating Centre in Occupational Health, University of South Australia, Magill Campus, St Bernards Road, Magill, Adelaide, SA 5072, Australia; harry.becher@unisa.edu.au; 2 Division of Psychiatry and Applied Psychology, School of Medicine, University of Nottingham, Nottingham NG8 1BB, UK; 3 Institute for Work and Health, Toronto, ON M5T 3M7, Canada; PSmith@iwh.on.ca; 4 Dalla Lana School of Public Health, University of Toronto, Toronto, ON M5T 3M7, Canada; 5 Department of Public Health and Preventive Medicine, Monash University, 553 St Kilda Road, Melbourne, VIC 3004, Australia; 6 Institute of Occupational, Social and Environmental Medicine, Centre for Health and Society, Faculty of Medicine, University of Düsseldorf, Universitätsstraße 1, 40225 Düsseldorf, Germany; Jian.Li@uni-duesseldorf.de

**Keywords:** circulatory diseases, Psychosocial Safety Climate, Demand-Control, effort-reward imbalance, psychosocial risks

## Abstract

Circulatory diseases (CDs) (including myocardial infarction, angina, stroke or hypertension) are among the leading causes of death in the world. In this paper, we explore for the first time the impact of a specific aspect of organizational climate, Psychosocial Safety Climate (PSC), on CDs. We used two waves of interview data from Australia, with an average lag of 5 years (excluding baseline CDs, final *n* = 1223). Logistic regression was conducted to estimate the prospective associations between PSC at baseline on incident CDs at follow-up. It was found that participants in low PSC environments were 59% more likely to develop new CD than those in high PSC environments. Logistic regression showed that high PSC at baseline predicts lower CD risk at follow-up (OR = 0.98, 95% CI 0.96–1.00) and this risk remained unchanged even after additional adjustment for known job design risk factors (effort reward imbalance and job strain). These results suggest that PSC is an independent risk factor for CDs in Australia. Beyond job design this study implicates organizational climate and prevailing management values regarding worker psychological health as the genesis of CDs.

## 1. Introduction

Circulatory Diseases (CDs), are a group of disorders of the circulatory system including myocardial infarction, stroke, angina and hypertension (as defined by the World Health Organization, International Classification of Diseases and Related Health Problems (ICD)-tenth revision) [1]. CDs are among the greatest health risks in the world [2], causing more deaths than any other single cause, accounting for approximating 30% of deaths annually worldwide [3]. Among working age populations 10–20% of all causes of CDs deaths are work related [4]. Despite some major improvements in circulatory health via public health interventions, CDs continue to grow as a global pandemic. This widespread health impact has a correspondingly large impact on workplace productivity; CDs have been classified as the single greatest cause of workplace productivity loss in the world [5,6]. In addition to commonly-known circulatory disease risk factors such as smoking and obesity [5], work-related psychosocial risk factors and job stress have been established as predictors of CDs [3,6,7,8,9,10]. 

Most CDs studies have focused on job design frameworks such as the Job Demand-Control [11], or the Effort-Reward Imbalance model [12] to explain work stress related CDs. These models have focused on proximal work-related psychosocial risk factors (i.e., job design characteristics that are harmful to health), yet the root cause may be more contextual and relate to features of the organizational climate that potentially shape these harmful job characteristics. Focusing on the “causes of the causes” has been identified as a key focus for future research [3]. In this research, we contextualize CDs as a work-related health problem that may be predicted by organizational factors further upstream. We use Psychosocial Safety Climate (PCS, i.e., the organizational climate for worker psychological health) theory to frame the study. Under PSC theory working conditions are determined by the prevailing management values concerning worker psychological health. To date there is much evidence linking PSC to work conditions and health outcomes [13,14,15] but no studies have explored the link between this specific aspect of organizational climate and CDs. 

The aim of this study is to determine whether PSC is a predictor of future employee CDs. 

### 1.1. Work Stress Theories and CDs

There is a long history of research linking work factors to CDs under work stress theoretical frameworks. Effort-Reward Imbalance theory posits the primary cause of job stress and related health effects is an imbalance between excessive efforts and insufficient rewards [12,16]. The Job Demand-Control theory [11] posits that the health of workers is determined by the level of job demands they experience, in combination with levels of control, such as decision authority and skill discretion. 

The potential increased risk of CDs associated with job stress has been examined using the Effort-Reward Imbalance and Job Demand-Control models across a range of studies and populations [6]. Evidence shows that effort-reward imbalance is linked to CDs. An 11-year longitudinal analysis of the Whitehall II data revealed that those in high effort-reward imbalance jobs are 26% more likely to develop coronary heart disease than their peers [17]. A 24-year longitudinal analysis of Finnish workers, revealed that workers with high effort-reward imbalance were 140% more likely to develop CDs than their peers [18]. Effort-reward imbalance has been recently confirmed as an important increased risk factor for CDs, using large pooled data from 11 European cohort studies (RR = 1.16; [19], over and above established risks such as long working hours (Relative Risk [RR] = 1.39) [20] and job insecurity (RR = 2.00) [21]. The overall burden of this increased risk is considerable. 

There is also a strong literature linking Job Demand-Control job strain (i.e., high job demands and low control) to CDs, across major demographics and over time [3,22,23] to non-fatal and fatal myocardial infarction, where, after adjustment for sex and age, the hazard ratio for job strain versus no job strain was 1.23, with the effect higher in published (HR = 1.43) than unpublished (HR = 1.16) studies [23]. Job strain is associated with an increased risk of ischemic stroke [24], an increased risk of non-fatal myocardial infarction in women [25] and research has found support for both job strain and effort-reward imbalance as independent risk factors for stroke [26].

Workers experiencing chronic work stress have increased blood pressure [27], even when they are not at work [28]. The mechanism for this effect is thought to be a combination of hyper-reactivity of the sympathetic nervous system, along with reduced vagal tone—a symptom of reduced activity of the parasympathetic nervous system [28]. A systematic review found support for the effects of both Job Demand-Control and Effort-Reward Imbalance Models on blood pressure level and hypertension in approximately half of the studies reviewed [29].

The problem with focusing on Effort-Reward Imbalance and Job Demand-Control models, fundamentally job design theories, is that they do not address a potential origin of the problem, Psychosocial Safety Climate. 

### 1.2. Psychosocial Safety Climate Theory

Psychosocial Safety Climate refers to “organizational policies, practices and procedures for the protection of worker psychological health and safety” [30] (p. 580). PSC is largely determined by management values and practices and organizational systems that enable communication and participation, in prevention, identification and resolution of work stress related issues. In high PSC contexts managers are concerned for worker health and wellbeing and consequently design jobs that have manageable demands and adequate resources [15,30,31]. Low PSC workplaces are characterized by senior management values that prioritize short-term productivity over the psychological health of employees and jobs may be designed with unmanageable psychological and emotional demands [14]. Since PSC predicts the way jobs are designed, it is theoretically a precursor to the job design stress theories and has been shown empirically to predict effort-reward imbalance [32] and Job Demand–Control job strain [33]. 

Psychosocial Safety Climate also predicts psychological health outcomes such as depression [34,35], psychological distress [15,34] and emotional exhaustion [15]. Yet, CDs have not yet been investigated as an outcome of PSC. There is limited evidence available concerning the predictive power of PSC on physical health. Longitudinal designs are better suited to teasing out causal effects. The current study addresses a gap in the literature by examining the link between PSC and future CDs over a subsequent four to six years after initial measurement. Since PSC can negatively predict a range of risk factors for work stress, including those embodied in Effort-Reward Imbalance and Job Demand-Control theories we propose a hypothesis, that PSC negatively predicts future CDs after controlling for effort-reward imbalance and Job Demand-Control job strain. 

## 2. Materials and Methods

### 2.1. Participants

Participants were interviewed using Computer Assisted Telephone Interviewing as part of the Australian Workplace Barometer (AWB) project, a national surveillance survey of psychosocial risks in Australian workplaces. We used a subsample of the wider AWB study, including participants with data at two times points on average 5 years apart, excluding, self-employed and missing data on health outcome measures.

The final sample comprised 1223 participants who were free from any CDs at Time 1, 545 (44.6%) males and 678 (55.4%) females, aged between 18 and 73 (median = 47 years) at Time 1. Their education status was diverse with 35.8% holding a bachelor degree or higher, 29.4% with a certificate or diploma, 8% trade/apprenticeship, 17.7% left school after age of 16, 9.1% left school at 16 years or less. The median annual income was 50 to 60 thousand AUD. The participants were located in three different Australian states: South Australia (*n* = 428), Western Australia (*n* = 439) and New South Wales (*n* = 356). Time 1 data collection was conducted in 2009 in NSW and WA and 2010 in SA. Time 2 data was collected in 2014–2015 across all three states. All subjects gave their informed consent for inclusion before they participated in the study. The study protocol was approved by the University of South Australia Human Research Ethics Committee (approved 17 June 2009, protocol number 0000024586).

### 2.2. Measures

Information was collected on participants’ age, gender, socioeconomic status and education level, which were included as covariates in the analyses as in other CDs research (e.g., [36]). 

Psychosocial Safety Climate was measured using the PSC-12, a 12-item questionnaire consisting of the four sub-scales each of which have three items [37]. The subscales and example items are; management commitment, e.g., “In my workplace senior management acts quickly to correct problems/issues that affect employees’ psychological health”; management priority, e.g., “Senior management considers employee psychological health to be as important as productivity”; organizational participation, e.g., “Employees are encouraged to become involved in psychological health and safety matters” and organizational communication, e.g., “There is good communication here about psychological safety issues which affect me.” Responses are scored on a 5-point Likert scale from 1 (strongly disagree) to 5 (strongly agree). Since the subscales are highly correlated for practical purposes we added all the scales together to form a global measure, α = 0.94. Psychosocial Safety Climate benchmarks used in this study, developed by Bailey and colleagues [14], were PSC low (≤37), moderate (37.01–40.99) and high (≥41). 

Circulatory diseases were used as the outcome measure as in other occupational medicine research [38] using questions from the World Health Organization Health and Work Performance Questionnaire [39]. Participants were asked whether “in the past two years have you consulted a health professional with regard to chest pain, or any other cardiovascular related health problem—such as myocardial infarction; angina; stroke; or hypertension?” and if so, what diagnosis was returned and the four disease categories were listed. CVD was dummy coded as 1 (circulatory diseases diagnosed) or 0 (no doctor visit or no circulatory disease diagnosed). We ruled out “other” diagnoses mentioned such as heart murmur, stress, blood clot, no problem. 

Effort-reward imbalance was measured using the ratio of effort to reward. For convenience, extrinsic effort was measured using five items from the psychological demands subscale of the Job Content Questionnaire (JCQ, [40]), with responses on a four-point Likert scale with responses ranging from 1 (*strongly disagree*) to 4 (*strongly agree*). Higher scores represent a greater amount of perceived effort by the worker, α = 0.68. Rewards were measured based on 4 items from the Effort-Reward Imbalance Scale [12]: “Considering all my efforts and achievements, I receive the respect and prestige I deserve at work.” Higher scores represent a greater amount of perceived organizational rewards received by the worker, α = 0.68. Effort-reward imbalance was calculated using the ratio method as recommended by Effort-Reward Imbalance theorists and this formulation has construct validity. The formula for this calculation is $\frac{\mathrm{Effort}}{\mathrm{Rewards}\;\times \;1.25}$. 

Job Demand-Control job strain was assessed using combinations of job demands and control. Job demands was the same measure as “effort” described above, assessed with the five item psychological demands subscale of the JCQ [40], with responses on a 4-point Likert scale with responses ranging from 1 (strongly disagree) to 4 (strongly agree). Higher scores represent a greater amount of perceived demand by the worker, α = 0.68. Job control was assessed from the Job Content Questionnaire ([40], www.jcqcenter.org) subscales, skill discretion (six items, e.g., My job requires a high level of skill; α = 0.75) and decision authority (three items, e.g., “My job allows me to make decisions on my own”; α = 0.73). Responses are on a 4-point Likert scale from 1 (strongly disagree) to 4 (strongly agree). We used the quartile-based job strain as recommended because of their greater sensitivities than the median-based job strain definition with no significant changes in specificities. This yielded five distinct groups—low strain, high strain, passive work, active work and midpopulation. The Job Demand-Control job strain measure used here was assigned 1 (high strain) and 0 (other groups) and three other dummy variables were entered in the model simultaneously (e.g., 1 (active work) and 0 (other groups) and so on). 

### 2.3. Statistical Analyses

We used SPSS 24 software for all analyses (IBM Corp., 2016, Armonk, NY, USA). For hypothesis testing since the outcome measure was binary we used binary logistic regression. After removing baseline cases of CDs (*n* = 97) we regressed Time 2 CDs on the demographic covariates and PSC (Model 1) and entered the work environment measures as controls (Model 2). 

## 3. Results

### 3.1. Correlations between Measures

As shown in Table 1, of the demographic variables (age, gender, education, income) only age was significantly associated with CDs at Time 2. Of the work measures, PSC was significantly negatively related to CDs at Time 2. 

### 3.2. Incidence Rate of CDs

Over the 5 years period, 98 new CDs cases occurred among 1223 participants who were free from any CDs at Time 1 (cumulative incidence rate = 8%). We conducted some preliminary incidence tests of CDs by PSC benchmarks. Comparisons between low, moderate and high PSC environments demonstrate that those in high PSC environments exhibited lower rates of overall CDs after an approximate five year time lag (see Table 2). Participants in low and moderate PSC environments were more likely (59% and 45% more, respectively) to develop CDs than those in high PSC environments. The sensitivity analyses, using different levels of PSC, demonstrated a higher level of CDs in participants working in low PSC (high risk) work environments. 

As shown in Table 3, Model 1, we regressed new incidences of CDs on demographics and PSC. The demographics age and education at Time 1 were related to CDs at Time 2; older workers were more likely to experience new CDs as were workers with less education. PSC was significantly negatively related to CDs; workers from organisations with lower levels of PSC reported higher levels of new incidences of CDs than those from organisations with higher level of PSC (B = −0.02, SE = 0.01, *p* = 0.04, OR = 0.98 with the 95% confidence interval (0.96–1.00).

In Model 2 we controlled for the significant demographics (age and education) from Model 1 and also controlled for job design stressors (job strain and effort-reward imbalance). PSC was significantly related to future CDs with effects the same as in Model 1. Our hypothesis that PSC predicts future CDs (over and above effects due to demographics and job design factors (effort-reward imbalance and Job Demand-Control job strain) was supported. 

## 4. Discussion

Studies which have neglected to assess PSC may have underestimated the effect of the work context on CDs. This research expands previous research that has linked task related psychosocial work conditions to CDs (e.g., [9]) by including a more distal organizational level factor, that is PSC. The aim of this research was to explore the relationship between PSC and CDs. We also explored the relationship between PSC and CDs before and after adjustment for known job design risk factors for CDs, effort-reward imbalance and Job Demand-Control job strain. We used a two-wave longitudinal sample to show the causal nature of the effect of organizational factors on CDs in workers. Furthermore, an average 5 years gap between the first and the final rounds of data collection allowed us the opportunity to examine the longer term effect of psychosocial risks on a chronic and ongoing health problem (i.e., CDs). This is an important contribution to the literature, as many studies either only present a cross-sectional correlation [41], providing no evidence of causation, or include time lags as short as one year [42] which is insufficient to measure the onset of many chronic diseases. 

Logistic regression showed that PSC is significantly negatively related to higher CDs risk (OR = 0.98, 95% CI 0.96–1.00). This risk remained, after additional adjustment for job strain and ERI measures. Work job design factors, effort-reward imbalance and job strain were not significant contributors to future CDs. This is somewhat at odds with much previous research showing the detrimental effect of Job Demand-Control job strain and effort-reward imbalance on CDs, for instance in relation to Dragano et al.’s [19] multi-cohort finding but perhaps not surprising since our sample size was much smaller (cf., 90, 164) and the prediction time span was smaller (cf., 9.8 years). Nevertheless, the research demonstrates that a climate for psychological health and safety predicts future circulatory diseases. Workers who believe that their employers are not prioritizing their mental health evident through supportive systems and processes are more likely to experience circulatory diseases over the next five years. 

### 4.1. Practical Implications

Psychosocial Safety Climate is a reflection of the priorities and practices of senior management in relation to worker psychological health within an organization and therefore presents an ideal intervention point for those seeking to address the workplace psychosocial factors relating to CDs. 

In the UK alone, a 1% reduction in CDs risk is estimated to prevent 25,000 CDs cases per year and save €40 million per year [43]. Assuming similar PSC rates to Australia, if workers in low and moderate PSC workplaces had their CDs incidence reduced to that of workers in high PSC workplaces, this would represent around 40% decrease in CDs risk, or approximately €1.6 billion per year in UK terms. Workplace interventions to improve PSC could potentially reduce CDs risk substantially, saving billions of dollars in developed countries around the world. 

This study provides policy makers with additional evidence of the harm caused by psychosocial risks in the workplace. Given the substantial body of evidence demonstrating the important role that job characteristics play in the aetiology of CDs and the evidence that PSC precedes work conditions (e.g., [33]) and the link between PSC and CDs shown here, there is a drastic need for organizational intervention research to determine whether psychosocial risk prevention particularly focusing on PSC reduces CDs in workers, potentially saving lives, improving wellbeing and increasing productivity. Policy makers should consider psychosocial risk management as an additional tool in the public health campaigns aimed at reducing CDs. Businesses that wish to improve organizational health should consider a PSC intervention to reduce CDs onset in workers.

### 4.2. Limitations

Our study analysed PSC at the individual level, despite its conceptualization as an organizational level construct. Population-based sampling provided a representative sample of Australian workers from all major industries, occupations and demographic groups. However, it also provides fewer identifiable organizations with sufficient group sizes for multilevel analysis [44]. Given that CDs only occurred in a small proportion of the population and the population-based sampling technique used, analysing PSC at the organizational level would have reduced the power of the analysis too severely. As such, the likelihood of a Type II error would be too high, so an individual level analysis of PSC was used. It is possible that the assumption of independence of the data used was violated, as approximately 20% of the participants belonged to the same organization. We justified analysis at the individual level based on previous research demonstrating that PSC has some individual level properties separate from organizational level influences [14,30]. Another limitation goes to the measurement of CDs. In our study, the incident cases of CDs were based on self-report. Though register data (such as hospitalization records) are generally preferred, it has been shown that self-reported CDs have reasonable sensitivity and specificity, with acceptable agreement to medically certified records [45]. Another limitation is that only non-fatal CDs were considered because the measure was based on self-report. Therefore, we lack information on deaths during the follow-up period.

Although not the main objective of this study, we observed that ERI and JD-C job strain were not correlated with CDs—this result should be viewed with caution because of the limited sample size and the healthy worker effect, whereby those with high levels left the sample reducing potential effect sizes. Compared to our very initial sample of 3030, the proportion of employees in high strain jobs was 21% whereas in our matched sample over 5 years it was around 16%. Our results may be at variance with other studies, due to measures used, relatively short follow-up time (5 years) for research on cardiovascular epidemiology and the general working population sample. Our outcome measure of circulatory diseases was not optimal compared to more strict definitions used in cardiovascular disease research but given the small sample size and few incident cases for each cardiovascular disease type we had to combine them together.

## 5. Conclusions

This longitudinal research, in Australia, showed that circulatory problems newly diagnosed by a doctor, could be predicted over a five year period by knowing about PSC levels within organizations. Understanding the association between an organization’s PSC and the CDs risk borne by its workers provides an evidence basis, for organizational personnel (managers, HR, OHS and unions) to consider improvements in PSC to improve work conditions and reduce CDs and improve productivity (e.g., less time off due to illness) and more broadly for policy makers to consider potential legislative requirements and responses to target organizations to reduce CDs risk and public health costs through improving PSC. 

## Figures and Tables

**Table 1:** Intercorrelations between study variables.

Variables	1	2	3	4	5	6	7
Age T1							
Gender T1	0.03						
Education T1	−0.04	0.05					
Income T1	0.17 ***	−0.43 ***	0.27 ***				
Job Strain T1	−0.01	0.09 ***	−0.04 *	−0.05			
Effort-Reward Imbalance T1	0.00	0.10 ***	0.11 ***	0.05	0.37 ***		
Psychosocial Safety Climate T1	−0.00	0.02	−0.04	−0.04 *	−0.35 ***	−0.25 ***	
CDs T2	0.11 ***	0.00	−0.07	0.02	−0.00	0.02	−0.06 *

Note: *** *p* < 0.001; * *p* < 0.05. T = Time. *n* = 1223.

**Table 2:** PSC benchmarks and CDs incidence.

PSC Time 1	Number of Participants	Participants with CDs at Time 2	% with CDs at Time 2	Average Higher Incidence Compared to High PSC
Low	365	41	11.23%	59%
Moderate	97	10	10.30%	45%
High	663	47	7.08%	

Note: Low PSC ≤ 37; Moderate PSC 37.01–40.99; High PSC ≥ 41. *n* = 1223 (history of CDs removed).

**Table 3:** Predicting Circulatory Diseases at Time 2.

Models	Variables	B	SE	Wald	Sig.	Odds Ratio	Low CI	High CI
Model 1	Constant	−4.30	0.74	33.31	0.00	0.01	0.00	0.06
	Age Time 1	0.04	0.01	18.62	0.00	1.05	1.02	1.06
	Gender Time 1	0.07	0.22	0.11	0.74	1.08	0.70	1.65
	Education Time 1	−0.13	0.06	5.25	0.02	0.88	0.78	0.99
	Income Time 1	0.08	0.05	2.45	0.12	1.09	0.98	1.19
	Psychosocial Safety Climate Time 1	−0.02	0.01	4.22	0.04	0.98	0.96	1.00
Model 2	Constant	−3.08	0.98	9.81	0.00	0.05	0.01	0.31
	Age Time 1	0.04	0.01	12.99	0.00	1.04	1.02	1.06
	Education Time 1	−0.13	0.06	4.84	0.03	0.87	0.78	0.99
	Effort-Reward Imbalance Time 1	0.51	0.47	1.18	0.28	1.66	0.66	4.18
	JCQ Job Strain Time 1	−0.47	0.45	1.08	0.30	0.62	0.26	1.51
	Psychosocial Safety Climate Time 1	−0.02	0.01	4.34	0.04	0.98	0.96	1.00

Note: PSC was entered as a continuous measure as was effort-reward ratio. Job strain was entered with 3 other dummy variables. SE: standard error.

## Dedication

In honour of Harry Becher, his sharp mind and his passion for better work conditions.
